# Dissemination of sport-based psychosocial interventions in Europe: results from the EASMH project

**DOI:** 10.1192/j.eurpsy.2022.809

**Published:** 2022-09-01

**Authors:** G. Sampogna, M. Borgi, F. Cirulli, B. Collacchi, S. Cerino, V. Di Tommaso, S. Moliterni, A. Bichi, C. Barat, A. Pringle, S. Kivisto, I. Melenco, A. Oltean, J. Garside, A. Fiorillo

**Affiliations:** 1 University of Campania “Luigi Vanvitelli”, Department Of Psychiatry, Naples, Italy; 2 Istituto Superiore Sanità, Section On Neuroscience, rome, Italy; 3 ECOS, Ecos, Rome, Italy; 4 EPSI, Epsi, BRUSSELS, Belgium; 5 University of Nottingham, Psychiatry, Nottingham, United Kingdom; 6 University of Tempere, University, Tempere, Finland; 7 university of Costantia, Sport, romania, Romania; 8 Everton in the Community, Everton In The Community, London, United Kingdom; 9 University of Campania, Department Of Psychiatry, Naples, Italy

**Keywords:** rehabilitation, social integration, sport, Recovery

## Abstract

**Introduction:**

Among psychosocial interventions, recent studies have highlighted that sport-based interventions can positively impact on the long-term outcomes of patients with severe mental disorders, in terms of improving their quality of life and promoting social inclusion. Although sport-based interventions should be considered an effective strategy for promoting patients’ recovery, few data are available on their dissemination in the clinical routine care in Europe.

**Objectives:**

to evaluate the availability of sport-based psychosocial interventions in European countries.

**Methods:**

In the framework of the EU-Erasmus+, the European Alliance for Sport and Mental Health (EASMH) project has been funded. In order to evaluate the availability of sport-based interventions, an ad-hoc online survey, sent to national mental health centres, has been developed.

**Results:**

103 responses were obtained (49 from Italy, 31 from UK, 17 from Finland and 12 from Romania). The respondents were mainly psychiatrists working in community mental health centers. Sport-based interventions were frequently provided by mental health services, in particular in Italy, UK and Finland. While in UK and Finland sport-based interventions are commonly offered to all patients, in the other countries these are provided only by patient’s request. The most frequent types of sport practised were: running, football, volleyball, tennis and table tennis and basketball. Almost all respondents reported to not use a dedicated monitoring tool for evaluating the efficacy of those interventions.

**Conclusions:**

Sport-based interventions are not frequently provided in the routine clinical settings, although no monitoring tools are routinely adopted. The EASMHaims to fill this gap by disseminating good clinical practice related to sport-based interventions.

**Disclosure:**

No significant relationships.

